# An unusual case of vulvar schwannoma

**DOI:** 10.1186/s12957-015-0556-z

**Published:** 2015-04-08

**Authors:** Sung Taek Park, Han Myun Kim, Mi Kyung Shin, Ji Won Kim

**Affiliations:** Department of Obstetrics and Gynecology, Kangnam Sacred Heart Hospital, Hallym University College of Medicine, Seoul, 150-950 Korea; Department of Radiology, Kangnam Sacred Heart Hospital, Hallym University College of Medicine, Seoul, 150-950 Korea; Department of Pathology, Kangnam Sacred Heart Hospital, Hallym University College of Medicine, Seoul, 150-950 Korea; Department of Surgery, Kangnam Sacred Heart Hospital, Hallym University College of Medicine, 948-1, Daerim-1 Dong, Yeongdeungpo-gu, Seoul, 150-950 Korea

**Keywords:** Vulvar schwannoma, Vulvar mass

## Abstract

Schwannoma is a benign, solitary, slow-growing neoplasm of the peripheral nerve sheath. These tumors are rarely found in the external genital system, and only a few cases of vulvar schwannoma have been reported. Herein, we report a case of a vulvar schwannoma. A 37-year-old woman presented with a 3-cm-sized painless mass of the vulva which had been present for 3 years. Magnetic resonance imaging (MRI) of the pelvis showed an isolated finding of a 4.6-cm-sized round mass with a well-defined margin in the midline vulvar area. Simple excision of the tumor was undertaken, and histological examination with immunohistochemical testing demonstrated a vulvar schwannoma. Although benign schwannoma only rarely occurs in the vulva and other external areas of female genitalias, we suggest that it should be considered a differential diagnosis for patients that present a vulvar enlargement or mass. Simple surgical resection and follow-up is the most convenient treatment.

## Background

Schwannoma (also known as neurilemmoma) is generally a benign, solitary, nodular tumor of a peripheral nerve sheath. It can occur sporadically or in patients with neurofibromatosis. Its most common locations are in the head, neck, upper and lower extremities, posterior mediastinum, and retroperitoneum [1]. However, it is rarely found in the external female genitalia. According to our best knowledge, few cases of vulvar schwannomas have been reported in the literature. We describe a case of benign vulvar schwannoma and review of the literature.

## Case presentation

**Figure 1:**
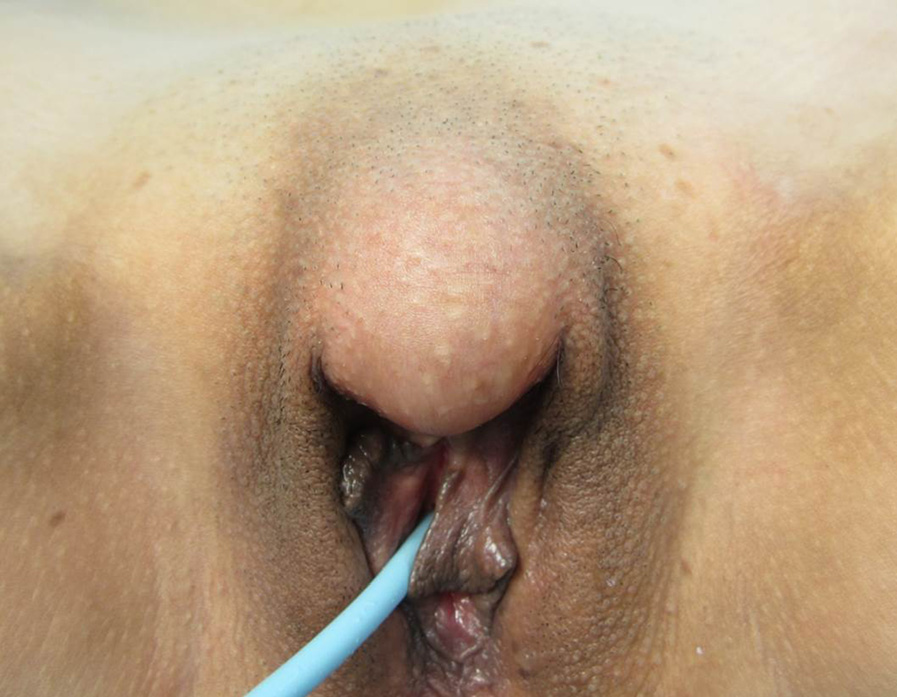
**Gross appearance of the vulvar schwannoma before surgical resection.** A subcutaneous non-tender, solitary tumor is seen in the upper vulvar area of both sides.

**Figure 2:**
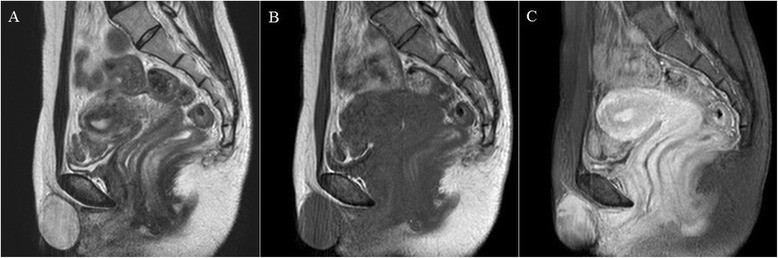
**Sagittal T2-weighted (A), T1-weighted (B), and post-contrast T1-weighted (C) magnetic resonance images.**

**Figure 3:**
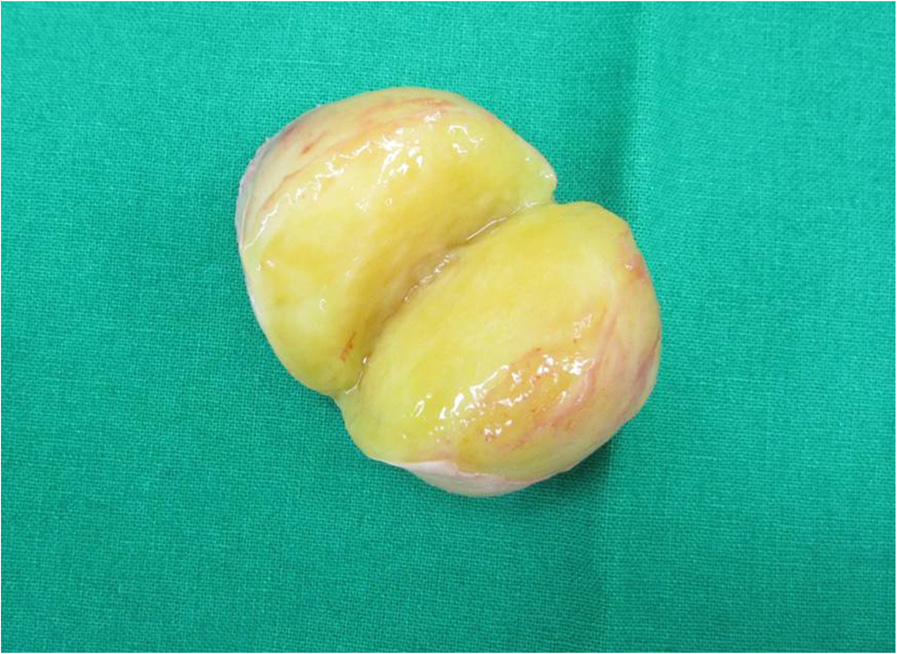
**A resected tumor.** The specimen was covered by fibrous capsule with its surface entirely pale yellow, soft, and homogenous.

**Figure 4:**
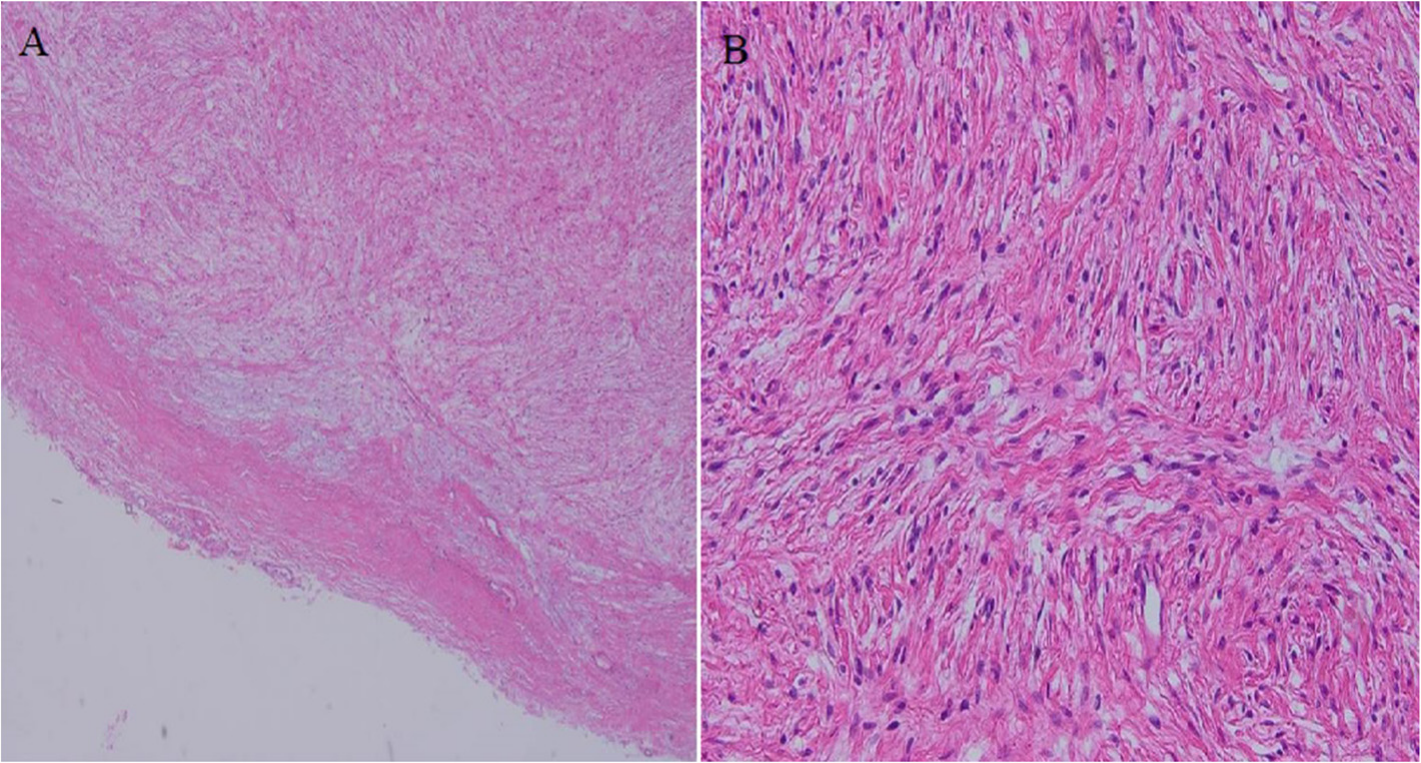
**A fibrous capsule with bland-looking spindle-shaped cells with ovoid or elongated nuclear in palisade pattern and a swirling pattern, forming Verocay bodies. (A)** HE stain, ×40. **(B)** HE stain, ×200.

**Figure 5:**
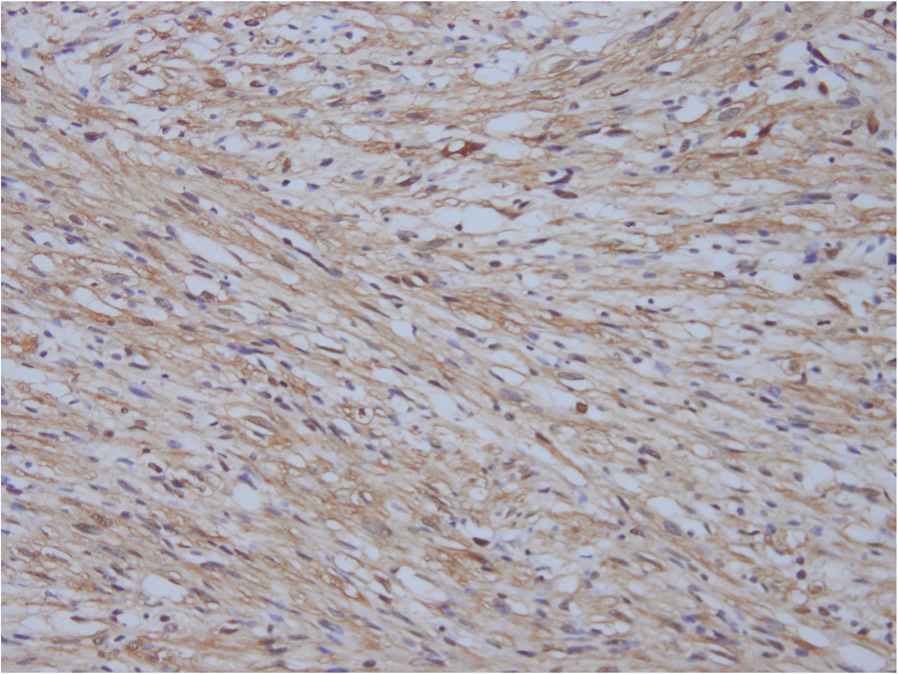
**Immunohistochemically, the spindle cells were diffuse positive for S-100 protein.**

A 37-year-old patient presented with a painless palpable vulvar mass, which was first noted 3 years prior to presentation, and its size had increased steadily ever since. There was no associated ulceration, bleeding, or pain, except for mild discomfort. There was no remarkable past medical, social, or familiar history of cancer or hereditary genetic disorders. On admission, vital signs (blood pressure, heart rate, respiration rate, body temperature) were within normal limits. The patient was in good general health and had no significant weight loss. On physical examination, there was a 3 × 2 cm sized solid subcutaneous mass located adjacent and superior to the clitoris, which was normal in size and shape, involving the upper midline portion of both vulva. It was an immobile and non-tender solitary mass with an irregular contour (Figure 1). The patient’s external genitalia, other than this lesion, were completely normal, and pubic hair distribution was feminine with no evidence of hyperandrogenism. The vulva and vaginal mucosa were normal, and there was no inguinal lymphadenopathy. Magnetic resonance imaging (MRI) of the pelvis presented a centrally located mass confined to the vulva with slight hypersignal intensity on T2-weighted imaging and low signal intensity on T1-weighted imaging. After injection with contrast agent, the mass was heterogeneously enhanced. The size of the lesion was about 4.6 cm, and the margin was well defined with a round shape (Figure 2). The results of laboratory tests and tumor marker analysis were normal. With the patient under general anesthesia, the tumor, located under the dermis and encapsulated, was excised with a clear margin. A histological examination was then performed. The resected specimen was a spherical solid tumor covered with a fibrous capsule. The cut surface was entirely pale yellow, soft, and homogenous with partially hemorrhagic foci and no necrosis (Figure 3). Under microscopic observation with hematoxylin and eosin (HE) staining, a fibrous capsule was identified with tumor cells and long spindle cells with ovoid or elongated nuclei, arranged a palisade pattern and a swirling pattern that formed Verocay bodies (Figure 4). No mitotic figures were identified. Immunohistochemical testing revealed that interweaving bundles of the spindle cells were strongly positive for S-100 protein (Figure 5). These features are consistent with a schwannoma. The patient was discharged 3 days after surgery without complication. No evidence of recurrence was noted after 8 months of follow-up.

## Conclusions

Schwannoma is a benign peripheral nerve sheath tumor, arising from Schwann cells in the myelin sheath of the peripheral nerves. Schwannoma is solitary, encapsulated, slow-growing tumor which is characteristically painless [1]. It is most frequently located in the head, neck, trunk, upper and lower extremities, posterior mediastinum, and retroperitoneum [2,3]. These tumors are rarely found in areas of the female genital system such as the clitoris, vulva, ovary, cervix, and round ligament, with less than ten case reports of schwannoma involving the vulva. The age range varies from 5 to 84 years, occurring most commonly in the age group of 20 to 40 [4,5]. Clinical symptoms may vary depending on which nerve is affected. But mostly, painless, immobile, and non-tender mass lesions are observed. Vulvar schwannomas are rarely multifocal [6]. They are usually benign and malignant schwannomas of external female genitalia are extremely rare, with less than 1% becoming malignant [7,8]. Differential diagnoses include Bartholin’s cyst, labial cyst, and mesenchymal tumors such as lipoma, liposarcoma, fibrosarcoma, and angiosarcoma [9]. The pre-operative diagnosis of a vulvar neoplasm is difficult; thus, biopsy may be required to exclude the possibility of malignancy and to decide upon an appropriate treatment. Also, radiologic imaging studies are important for characterization and differentiation between a benign and malignant vulvar mass. Vulvar schwannomas have previously been misdiagnosed as malignant tumors such as neurosarcoma, neurofibrosarcoma, and the malignant schwannoma [10]. Microscopic observation with HE staining can reveal the characteristic histologic appearance that can confirm accurate diagnosis. Histologically, the hallmark of schwannomas is a pattern of alternating Antoni type A and Antoni type B areas. Antoni type A tissue is composed of a tightly packed sheath of spindle cells arranged in palisade and swirling patterns. Antoni type B tissue is composed of loosely packed spindle cells and cells with small round nuclei. They also exhibit cystic degeneration, with Verocay bodies occurring as the tumor enlarges. Also, typical schwannoma features such as nuclear palisading and Verocay body-like structures occur without mitosis. A schwannoma must be distinguished from a neurofibroma, which is another benign tumor of the peripheral nerves with different histopathological characteristics. Microscopically, the presence of encapsulation, two types of Antoni areas, and diffusely strong immunostaining for S-100 protein distinguish schwannoma from neurofibromas [11]. The conventional variant is the most common type of vulvar schwannoma, but plexiform and ancient variant types also have been reported [2,9,12]. The most accurate diagnosis can be established through an immunohistochemical examination. A strong positive stain for S-100 protein appears in benign schwannomas. The malignant schwannomas are thought to be composed of dedifferentiated Schwann cells that had somewhat lost their capacity to synthesize S-100 protein [13]. The treatment of choice for benign schwannomas is complete excision, and prognosis is generally excellent following the operation. However, a relapse has been reported, which appears to have been a result of incomplete resection in a huge-sized schwannoma [14]. In our case, the mass was completely removed and to date, the patient is well, with no recurrence. Although benign schwannoma only rarely occurs in the vulva and other external areas of female genitalia, we suggest that it should be considered as a differential diagnosis for patients presented with vulvar tumors.

## Consent

Written informed consent was obtained from the patient for publication of this case report and any accompanying images. A copy of the written consent is available for review by the Editor-in-Chief of this journal.
